# Fast response and recovery polyaniline montmorillonite reduce graphene oxide polymer nanocomposite material for detection of hydrogen cyanide gas

**DOI:** 10.1038/s41598-023-32151-0

**Published:** 2023-05-18

**Authors:** Aparna Singh, Pukhrambam Dipak, Asif Iqbal, Anuradha Samadhiya, Shailendra Kumar Dwivedi, Dinesh Chandra Tiwari, Rajendra Kumar Tiwari, Kailash Nath Pandey

**Affiliations:** 1 School of Studies in Physics, Jiwaji University, Gwalior, India; 2 Vikrant University, Gwalior, India; 3 Department of Physics, School of Sciences, ITM University, Gwalior, India; 4 Applied Science and Humanities Department, IPS Group of Colleges, Gwalior, India; 5 DMSRDE, Kanpur, India

**Keywords:** Materials science, Nanoscience and technology, Physics

## Abstract

In the present work, we have developed a polymer based gas sensor. The polymer nanocomposites are synthesized by the chemical oxidative polymerization of aniline with ammonium persulfate and sulfuric acid. The fabricated sensor is able to achieve a sensing response of 4.56% for PANI/MMT-rGO at 2 ppm of hydrogen cyanide (HCN) gas. The sensitivity of the sensors PANI/MMT and PANI/MMT-rGO are 0.89 ppm^−1^ and 1.1174 ppm^−1^ respectively. The increase in the sensitivity of the sensor may be due to an increase in the surface area provided by MMT and rGO which provided more binding sites for the HCN gas. The sensing response of the sensor increases as the concentration of the gas exposed increases but saturates after 10 ppm. The sensor recovers automatically. The sensor is stable and can work for 8 months.

## Introduction

Hydrogen Cyanide (HCN) vapour is extremely hazardous to the living organism. HCN gas when inhaled it increases the intake level of oxygen by the cell^1–3^. The toxic level of the HCN gas is above 100 ppm and when exposed can kill a human being within 1 h^4^. Bhopal gas tragedy in 1984 killed 3,787 innocent people in a single night. This tragedy could have been prevented if some warning alarm system (gas sensor) is installed. The detection of trace amounts of toxic gasses (ammonia, dimethyl methyl phosphonate (DMMP), carbon monoxide, carbon dioxide, nitrous oxide, HCN) is important to prevent a fatal accident. Thus fabrication and development of electronic noses at the micro-and nano-level are needed. Fabrication of gas sensors using nanostructures increases the sensitivity of the sensors. The increase in surface area due to nanoparticles increases the binding sites of the gas. A sensor is a device when receives a stimulus it responds with an electrical signal^5–10^. Chemiresistance sensors work on the principle of change in resistance on exposure of gas. A standard sensor should satisfy the following characteristic features such as operation at room temperature, working in the ambient environment and no requirement of oxygen or air supply, no external stimulus is required, capacity to detect toxic gases at low concentration, high sensitivity and reproducibility, quick response and recovery, low cost and eco-friendly^11^.

Conducting polymer-based gas sensors has numerous advantages over the metal oxide sensors such as high sensitivity, short response time, operation at room temperature and can be tuned by the nature of the dopant. The sensitivity of the polymer base gas sensor is high due to the large surface-to-volume ratio, compact in size, lightweight and easy to integrate with existing electronic system^12^. Many researchers around the globe pay attention to the polymer nanocomposite material (organic–inorganic) due to their unique property such as increases flexibility, improved surface hardness and heat resistance (due to inorganic components)^8–10,13–15^. Yang et al.^16^ have reported the detection of HCN gas by quartz crystal microbalance (QCM) technique^16,17^.

Here we are reporting for the first time the detection of HCN gas by chemi-resistance method having fast response. In the present work, we have synthesized the Polyaniline/MMT-rGO nanocomposite by chemical oxidative polymerization. We used PANI as a sensing material in this study because of its stability, high sensitivity, good electrical conductivity, low cost, and ease of synthesis in the lab. rGO provides more binding sites due to its high surface area, thermal stability, and electrical conductivity. Montmorillonite (MMT) is used as a sensing material in the present study due to its high surface area, porous structure (which provides the large surface area), high adsorption coefficient, ease of tunable property (functionalization), environmental friendliness, and low cost. The synthesized polymer nanocomposite material is characterized by SEM, FTIR, and XRD. We can achieve a sensing response of 4.56% for PANI/MMT-rGO at 2 ppm of hydrogen cyanide (HCN) gas. The sensor recovers back to the baseline after every exposure of HCN. The sensor is stable and is working for the past 9 months successfully.

## Methods

### Materials

Aniline (C_6_H_5_NH_2_), sulphuric acid (H_2_SO_4_), hydrochloric acid (HCl), ammonium per sulphate ((NH_4_)_2_S_2_O_8_), potassium permanganate (KMnO_4_), sodium nitride (Na_3_N), hydrogen peroxide (H_2_O_2_) and hydrazine hydrate (H_6_N_2_O) are purchased from the Himedia. Ammonia (99.98%), acetone (99.9%), xylene (99%), benzene (99.9%), graphite and Montmorillonite (MMT) (CAS Number 1318-93-0) are obtained from Sigma Aldrich.

### Synthesis of reduce graphene oxide (rGO)

Graphene oxide (GO) was synthesized from graphite powder (Sigma-Aldrich) by using modified Hummers Method and further reduced by hydrazine hydrate to form reduced graphene oxide (rGO)^18–20^. Synthesized rGO is filtered by using Whatmann filter (125 micron), washed with deionized (DI) water, methanol and dried under vacuum.

### Preparation of PANI/MMT and PANI/MMT-rGO

0.5 M of aniline is added to distilled water of 50 ml and stirrered for 30 min. 0.5 m of H_2_SO_4_ is added to the above solution and stirrered for another 30 min. The functionalized MMT (0.5 g), 0.5 g of rGO are added and sonicated. Pre cooled solution of 0.5 M APS is added drop wise and kept for polymerization (8 h) below the 10 °C. The final nanocomposite is filtered using the Whatman filter paper (125 μm) and washed several times. Finally, the composite is washed with 10% of methanol to remove the unreactive chemical present in the composite. The polymer nanocomposite is dried at 60 °C. The PANI/MMT polymer nanocomposite material is prepared without adding rGO^21–23^ in the above process.

### Characterizations

X-ray diffraction (XRD) pattern is recorded on Rikagu (Model no Mini Flex 600) diffractometer using Cu-K_α1_ radiation with a wavelength 1.5406 Å in continuous scan mode at an acceleration voltage of 40 kV and current of 40 mA. To study the bonding nature in the polymer nanocomposite Fourier Transform Infrared Spectroscopy (FTIR) spectra is recorded (Ferkin Elmer Modal no. 105627 FT-IR). The surface morphology of flexible nanocomposite sensor film is analyzed by scanning electron microscopy (SEM). Transmission electron microscope (TEM) image is taken using TEM Jeol microscope operating at accelerating voltage of 120 keV.

### Gas sensing properties measurement

**Figure 1:**
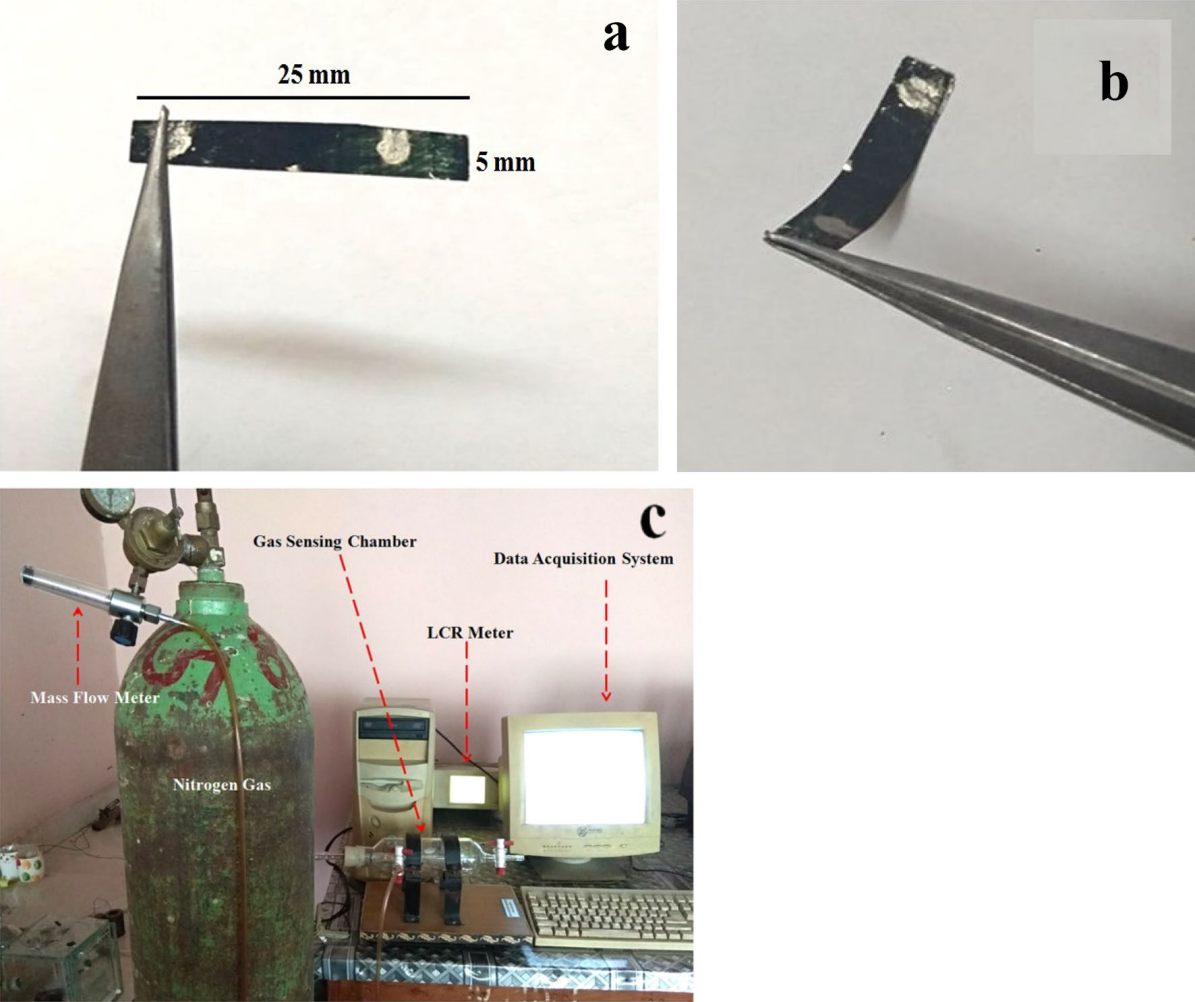
(**a**) Sensor (**b**) Showing the flexibility of the sensor, (**c**) gas sensor set up.

Thin films of polymer nanocomposite is deposited over the flexible transparency sheet (25 mm × 0.5 mm) by a drop-cast method and dried at 45 °C. Silver (Ag) paste is used to make electrodes for electrical measurements. The optical photographs of sensors flexibility is shown in Fig. 1a,b. The sensing activity of the sensor is conducted at simple home-made gas chamber of net volume 1 L. Different concentrations of HCN gas are introduced inside the chamber. The schematic diagram of the gas sensing assembly is shown in Fig. 1c.

Chemiresistance technique is used for the detection of HCN gas. The dynamic resistance of the sensor is measured when the sensor is exposed to HCN using an LCR meter (Hioki 3232) which gives final resistance and without HCN gives initial resistance. The sensing chamber is flushed with nitrogen gas before and after measurements.

The desired concentrations of gas are generated by static liquid distribution method^24,25^.

1 $$\mathrm{C}\left(\mathrm{ppm}\right)=\frac{22.4\mathrm{\rho TV}{^{\prime}}}{273\mathrm{MV}}\times 1000$$

where, C (ppm) is the desired target gas concentration, ρ is (g/mL) the density of the liquid (gas), V′ is the volume of liquid (μL), T temperature in Kelvin, M molecular weight of liquid (g/mol), and V volume of the chamber (L). Particular volume (μL) of analyte is injected into a chamber via a precision syringe. The gas chamber is flushed with the nitrogen (1000 sccm) gas before and after taking the reading. The sensor response, R%, is defined by^26^.

2 $$R \%=\frac{{R}_{f}{-R}_{i}}{{R}_{i}}\times 100$$

where, *R*_*i*_ is the initial resistance of the sensor, and *R*_*f*_ is the final resistance after exposure of HCN.

The sensitivity (S) of a sensor is defined by the slope of the graph drawn between sensing response versus concentration of the target gas:

3 $$\mathrm{S}=\frac{\Delta R}{\Delta C}$$

Here ∆R and ∆C are changes in sensor response and concentration of gas.

## Results

**Figure 2:**
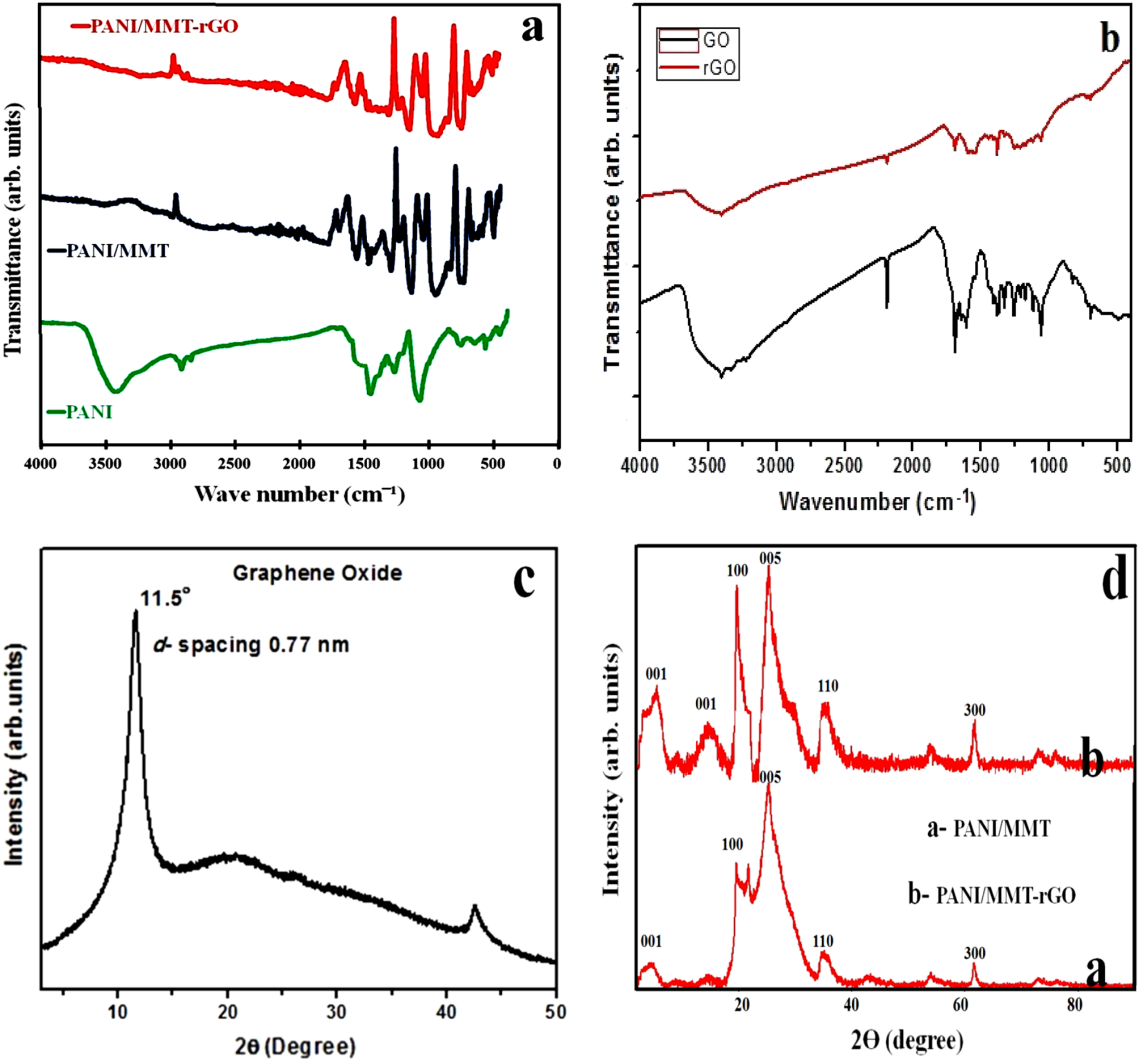
(**a**,**b**) FTIR of polymer composite and rGO, (**c**,**d**) XRD pattern of GO and polymer nanocomposite.

The graph of the FTIR studies of the GO, rGO, PANI, PANI-MMT and PANI/MMT-rGO is shown in Fig. 2a,b. The peaks of the FTIR studies are shown in Table 1. The characteristics peaks at 3398 cm^−1^, 1225 cm^−1^, 1054 cm^−1^, 1632 cm^−1^ correspond to O–H, C–OH, C–O and C=C stretching vibration of the rGO^20^. The characteristics peaks at 1112.12 cm^−1^ and 1088 cm^−1^ are due to C–H plane bending vibration. The peaks at 1306.15 cm^−1^ is due to C=N stretching mode, 1486.20 cm^−1^ and 1483.31 cm^−1^ peaks correspond to C=C stretching in the benzenoid ring and 1576 cm^−1^ is due to C=C stretching of quinoid in PANI^23,26,27^. The characteristics peaks at 1126 cm^−1^ and 1042 cm^−1^ are due to Si–O stretching, 917 cm^−1^ and 799 cm^−1^ are due to Al–OH stretching and 525 cm^−1^ and 465 cm^−1^ are due to Si–O bending vibration of the.

**Table 1:** FTIR study of GO, rGO, PANI, PANI/MMT and PANI/MMT-rGO.

	GO (cm^−1^)	rGO (cm^−1^)	PANI (cm^−1^)	PANI/MMT (cm^−1^)	PANI/MMT-rGO (cm^−1^)
O–H stretching vibrations	3398	3398	3398		
C–OH stretching vibration	1225	1225			1225
C–O stretching vibration	1054	1054			1054
C=C vibration	1632	1632			1632
C–H bending vibration			1112.12 & 1088	1112.12 & 1088	1112.12 & 1088
N–H stretching vibration			3393.50	3393.50	3393.50
C=C stretching benzenoid ring			1470.49, 1560.42	1470.49, 1560.42	1470.49, 1560.42
C = N stretching			1306.15	1306.15	1306.15
Si–O stretching				1126, 1042	1126, 1042
Al–OH stretching				917,799	917,799
Si–O bending mode vibration				525, 464	525, 464

MMT in composite^28^. The individual peaks of rGO, PANI and MMT are present in the PANI/MMT-rGO nanocomposite.

The XRD pattern of the rGO, PANI/MMT and PANI/MMT-rGO are shown in Fig. 2c,d. The peak at 2θ = 11.24° corresponds to the (001) plane of GO having an interlayer spacing of 0.77 nm, due to interlamellar groups trapped between hydrophilic graphene oxide sheets. The low-intensity peak at 2θ = 43.27° having (100) plane is due to rGO, thus confirming a random packing of graphene sheets in rGO^29–31^. The planes correspond to (001), (100), (005), (110) and (300) in the PANI/MMT are due to the MMT^30–32^. The plane (001) present at 15.5° in PANI/MMT-rGO composite is due to rGO. The individual peaks for rGO and MMT are found in the PANI/MMT-rGO polymer nanocomposite.

**Figure 3:**
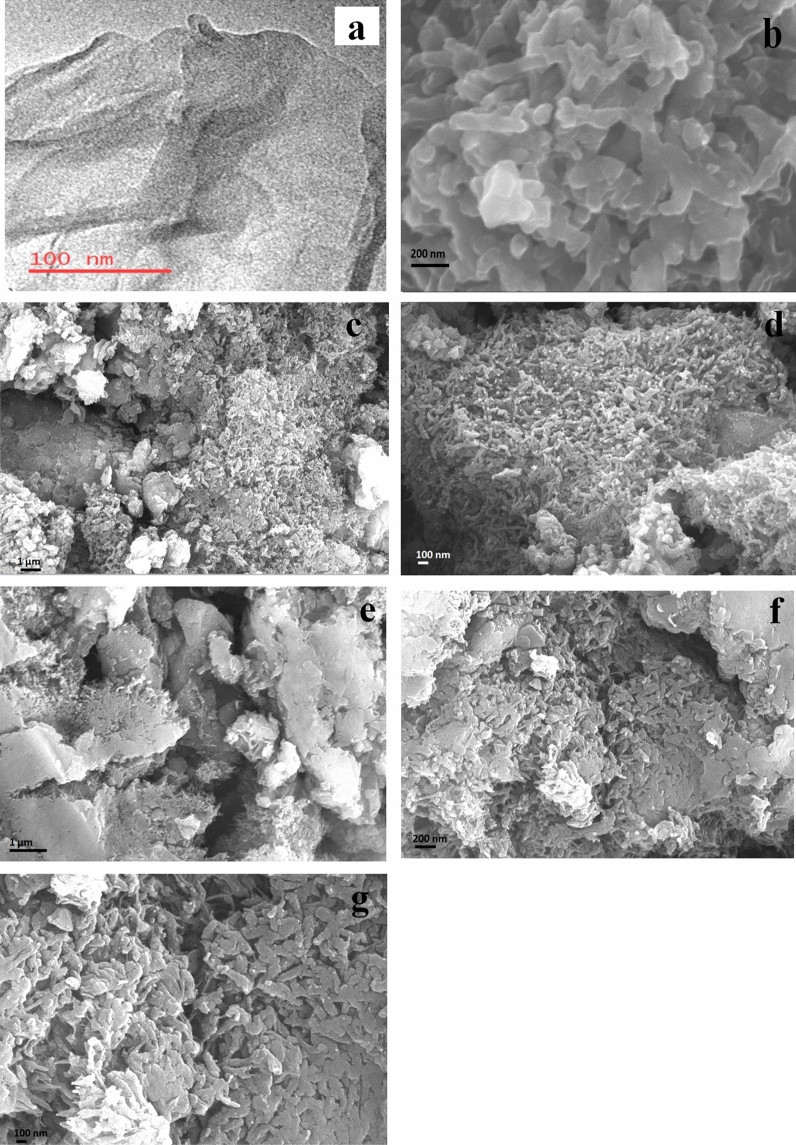
TEM image of (**a**) rGO, SEM images of (**b**) PANI, (**c**,**d**) PANI/MMT and (**e**–**g**) PANI/MMT-rGO.

The TEM micrograph of the rGO reveals the formation of a single layer sheet structure as shown in Fig. 3a. PANI has tube-like structures as seen in SEM of micrograph in Fig. 3b. The average length and diameter of the PANI are found to be 250 nm and 50 nm respectively. Figure 3c,d show the SEM micrograph of PANI/MMT. Here the PANI is deposited over the surface of the MMT. Thus it increases the surface area of the PANI. Figure 3e–g show.

The SEM micrographs of PANI/MMT-rGO at different resolutions. The SEM micrograph shows that the PANI is encapsulating over the rGO sheets and also deposited over the surface of MMT.

### Gas sensing properties

**Figure 4:**
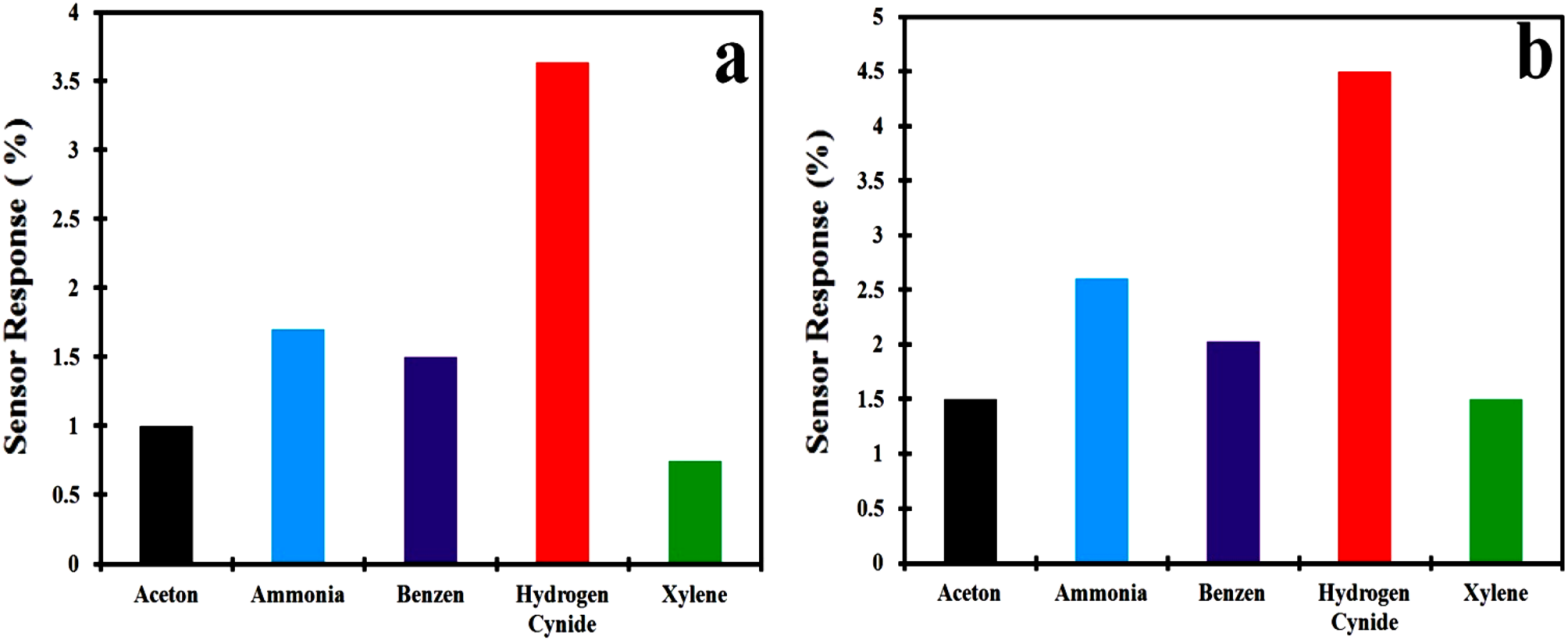
(**a**) PANI/MMT and (**b**) PANI/MMT-rGO response to different gases at 2 ppm.

The gas sensing study is carried out on the homemade chamber (1 L). 2 ppm concentration of different gases such as acetone, ammonia, benzene, hydrogen cyanide and xylene are introduced in the gas chamber containing the PANI/MMT gas sensor for the selectivity of the gas. The sensor is found to be more active to HCN having the sensing response of 3.5% as compared to the other gases as shown in Fig. 4a. Similarly, PANI/MMT-rGO sensor is exposed to different gases. Here we have found that the sensing response of the PANI/MMT-rGO toward the HCN is 4.56% as compared to other gases as shown in Fig. 4b. Thus both sensors have a good response toward the HCN gas.

**Figure 5:**
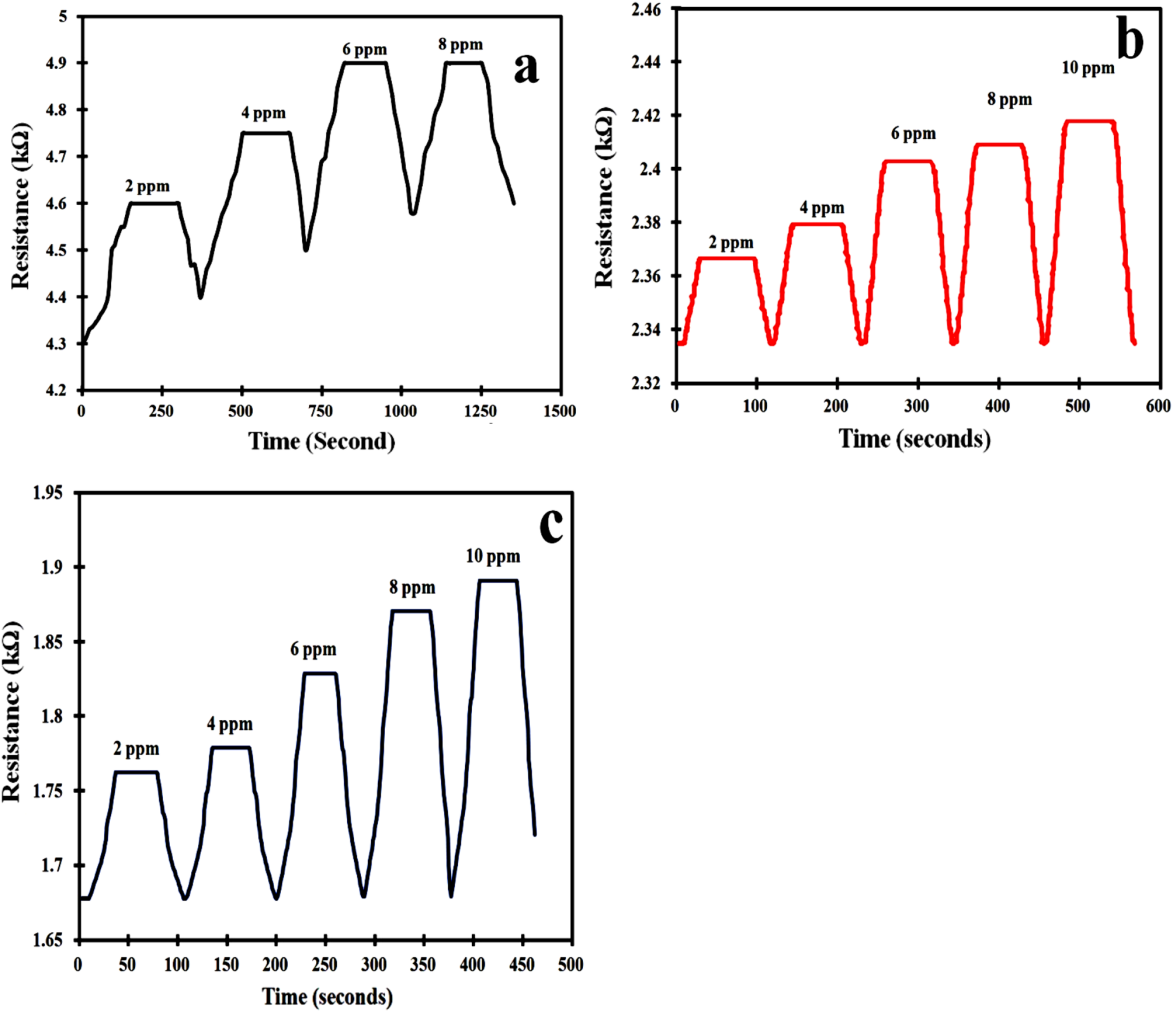
Sensor response to different concentration of HCN gas by (**a**) PANI, (**b**) PANI/MMT and (**c**) PANI/MMT-rGO.

The sensor made of PANI alone when exposed to the 2 ppm concentration of HCN has a sensing response is 0.045%. The sensing response (0.05%) is slightly increased when the concentration of the HCN gas vapor is 4 ppm but the sensor becomes saturated after 6 ppm as shown in Fig. 5a. The sensor (PANI) is not fully recovering to the initial baseline. This may be due to the fact that the HCN molecules are permanently bonded to the polymer chain^33^.

**Figure 6:**
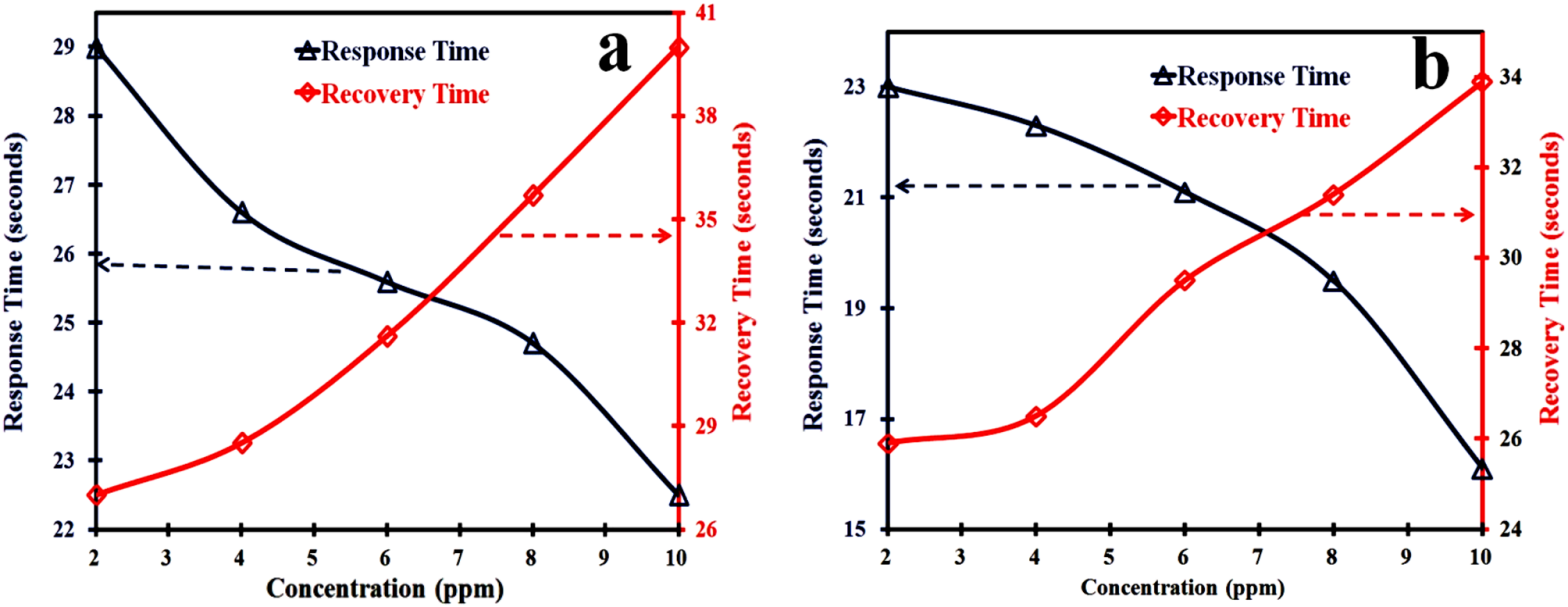
(**a**) PANI/MMT and (**b**) PANI/MMT-rGO : Graph between response time, recovery time with the concentration.

**Figure 7:**
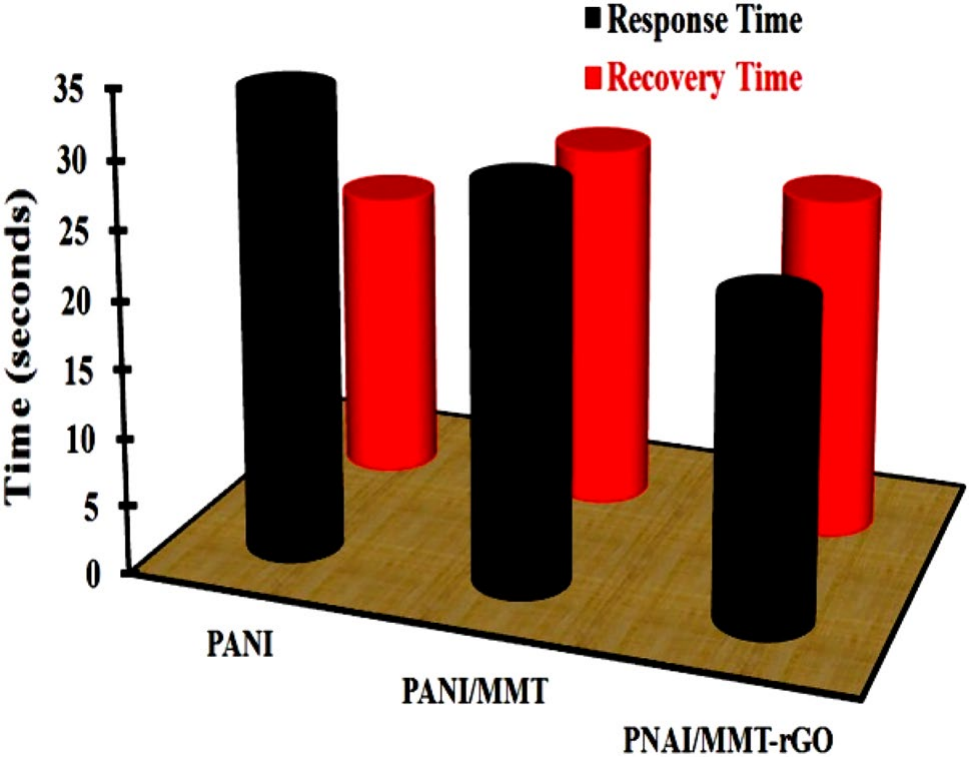
Comaprision response time and recovery time of sensors when exposed to 2 ppm HCN gas.

Both the sensors made up of PANI/MMT and PANI/MMT-rGO are exposed to 2 ppm, 4 ppm, 6 ppm, 8 ppm, and 10 ppm respectively. The sensing response of these sensors is calculated using Eq. (1) and shown in Table 2. The sensing response of the sensor becomes 3.5% (2 ppm) for PANI/MMT which has higher value as compared to the PANI alone (0.045%). The sensor response further increases as the concentration of the gas increase as shown in Fig. 5b. This increase in the sensor response (PANI/MMT) as compare to PANI sensors may be due to the increase in the binding sites provided by the MMT. The sensing response for PANI/MMT-rGO is 4.56% at 2 ppm, which is more than the PANI/MMT (3.5%) as shown in Fig. 5c. This increase in the sensor response is due to the increase in the surface area provided by rGO. In the case of graphene oxide, all the carbon atoms are available at the surface of the 2D sheet for binding with the exposed gas. Both the sensors PANI/MMT and PANI/MMT-rGO are recovering completely to the baseline. The response time of the sensor is defined as the time taken by the sensor to achieve 90% of the total sensor response. Figure 6a,b shows the response and recovery of PANI/MMT and PANI/MMT-rGO at different concentrations. The studies show that the PANI/MMT-rGO (29.5 s) sensor responded faster as compared to the PANI (34.5 s) and PANI/MMT (30.5 s) sensors. The sensor recovered automatically within 21 s for PANI/MMT and 25 s for PANI/MMT-rGO. Both the sensor (PANI/MMT and PANI/MMT-rGO) response and recovers less than a minute. In both the graph we observed that the response time of the sensor decreases as the concentrations of the HCN gas increase while the recovery time increases with the increases in concentrations. Figure 7 shows the graph of response and recovery time of all the sensors (PANI, PANI/MMT and PANI/MMT-rGO) exposed to the 2 ppm concentration of HCN gas.

**Table 2:** Sensors response towards HCN gas.

Concentration (ppm)	Sensing response (%)	
	PANI/MMT	PANI/MMT-rGO
2	3.50	4.56
4	4.75	6.00
6	7.00	8.40
8	8.10	10.25
10	9.25	11.75

**Figure 8:**
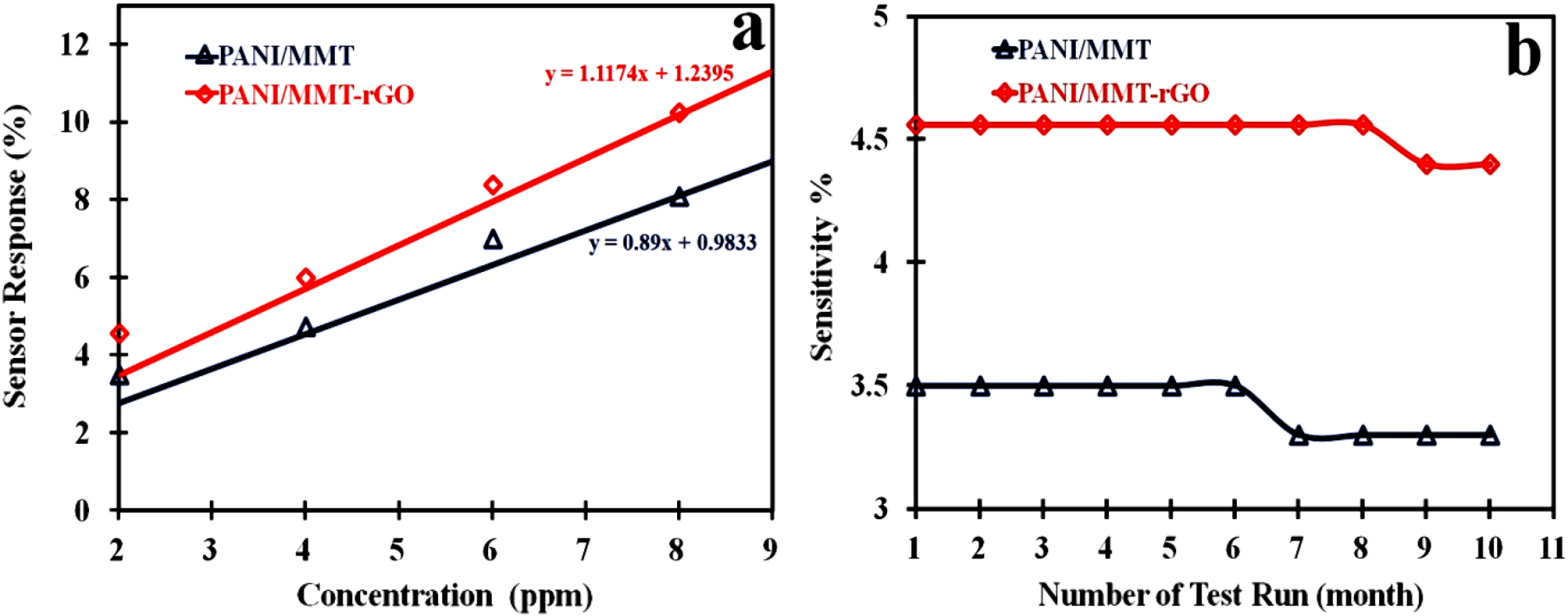
(**a**) Sensitivity Vs concentration and (**b**) Sensitivity Vs number of test run.

The sensing response versus concentration graph is shown in Fig. 8a. From the graph, we have calculated the sensitivity of PANI/MMT and PANI/MMT-rGO by using the Eq. (3). The sensors have a sensitivity of 0.89 ppm^−1^ for PANI/MMT and 1.1174 ppm^−1^ in case of PANI/MMT-rGO respectively. Table 3 shows the present work with the previously reported works.

**Table 3:** Comparison between the present and previously reported work.

Sensing Material	Lowest detection (ppm)	Response time (τ) (s)	Recovery time (τ) (s)	Sensitivity (ppm^−1^)	Authors
**PANI/MMT-rGO**	**2**	**29.5**	**25**	**1.1174**	**Present Work**
CuO	5	–	–	–	^23^
CuO	60	30	750	–	^15^
Keratin matrix with thiolated colloidal gold particles	150	–	–	–	^4^
Cobinamide-impregnated filter papers	5	–	–	–	^34^
Quantum dots graphene	5	90		0.2356	^35^
Dye-impregnated porous silicon photonic crystals	5	> 600	–	–	^36^

Both the sensors PANI/MMT and PANI/MMT-rGO are tested for 10 months by exposing 2 ppm concentration repeatedly, as shown in Fig. 8b. The sensing response of the sensor PANI/MMT remains constant for up to 6 months, but after which the sensing response becomes 3.25%. Similarly, the sensor PANI/MMT-rGO is stable up to 8 months and after that sensing response becomes 4.35%. Thus our sensors have a life span that varies from 6 to 8 months.

**Figure 9:**
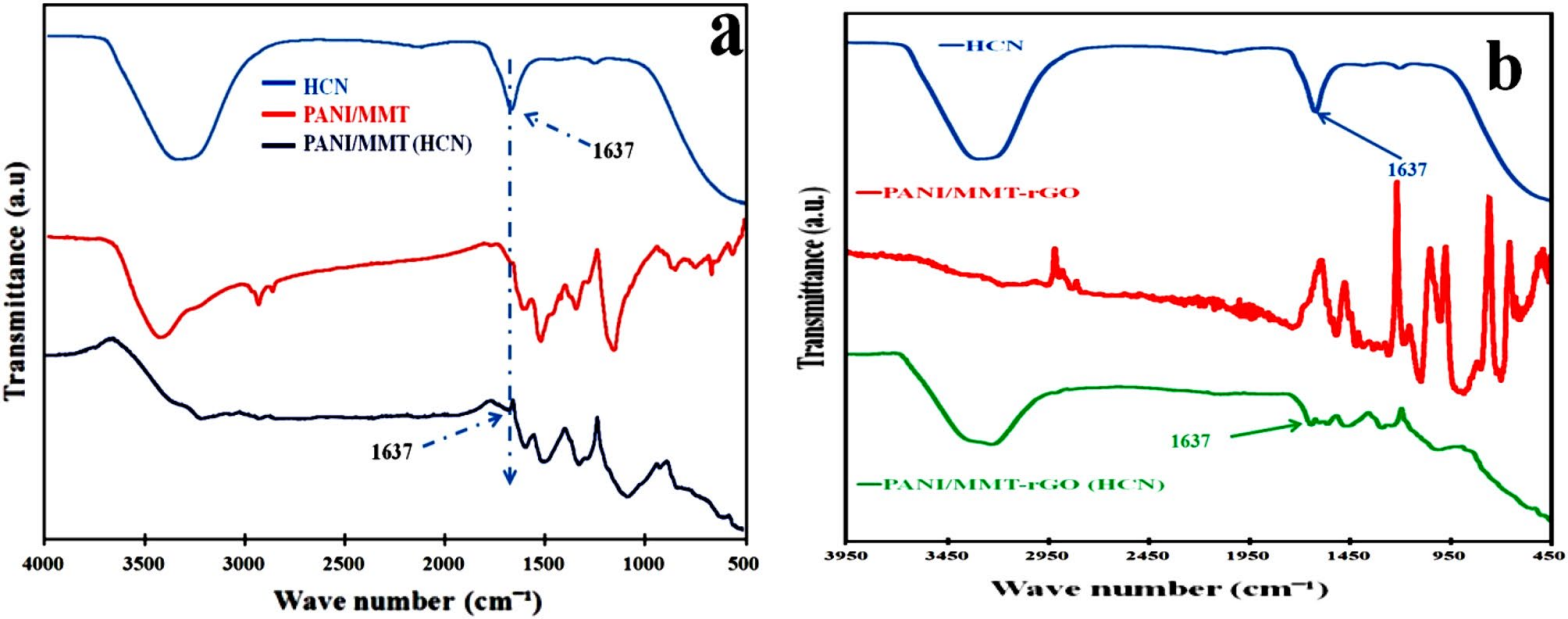
FTIR of (**a**) PANI/MMT and (**b**) PANI/MMT-rGO.

This decrease in the sensing response for both the sensors may be due to the decomposition of sensing material and the permanent binding of the HCN molecules to the sensing materials after repeated exposures. The FTIR studies of the sensing materials after 6 and 8 months show a minor peak at 1637 cm^−1^ in both PANI/MMT and PANI/MMT-rGO samples which are not present in the pristine PANI/MMT and PANI/MMT-rGO as shown in Fig. 9a,b.

**Figure 10:**
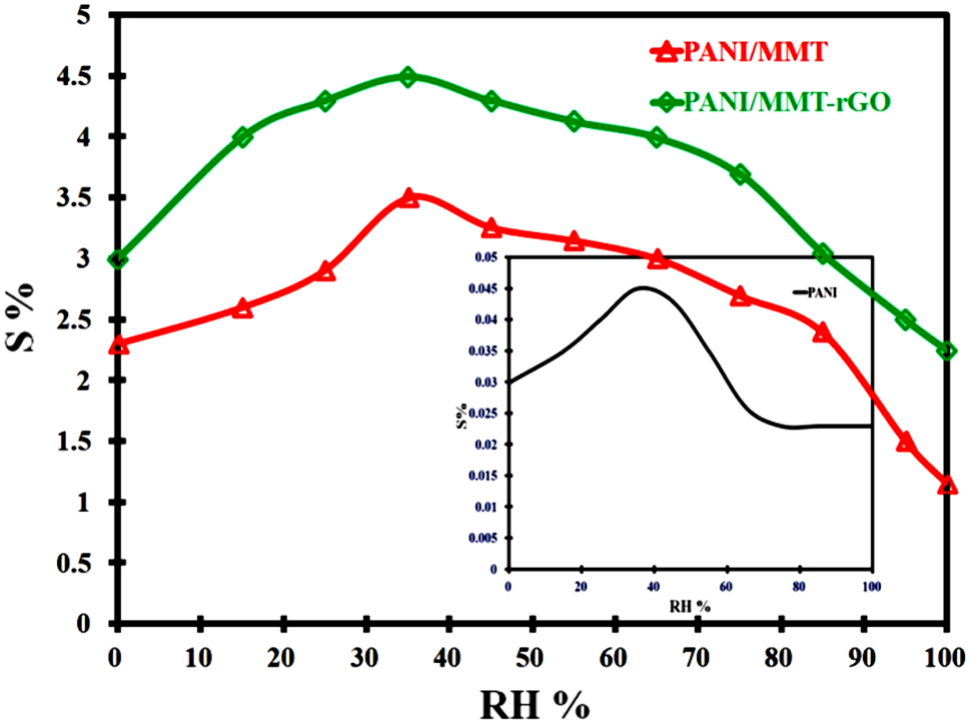
Sensor responses to HCN (2 ppm) starting from different RH values.

The fabricated sensors PANI, PANI/MMT and PANI/MMT-rGO are exposed to HCN gas at different relative humidity (RH). We have observed that the sensing response (S%) of the sensor increases as the RH increases but decreases after 40% of the relative humidity. Figure 10 shows the graph between the sensing responses with the RH when exposed to a 2 ppm concentration of HCN. The sensor resistance is changed by the presence of humidity. In the above Fig. 10, we have observed an increase in the sensing response of the sensor as the RH% increases, this may be due to the decrease in electrical resistance of the sensing material. Inside the sensing material, the pores are previously filled with dry air are now filled with a water molecule. But after an RH% value of 40%, the sensing response of the sensors decreases. This is due to the absorption of more water by the sensing materials causing an increase in resistance. It also increases the separation between the polymer chains, thus causing hindering of the electron hopping process. A similar phenomenon is also reported by Cavallo et al.^37^.

The interaction between the PANI and HCN may occur in two different ways, (a) H or N sites of the HCN with PANI or (b) N or H sites of the PANI with the HCN. In the HCN compound, the H atom is lacked electrons charge due to the high electro-negativity of the –CN group. During the interaction between the PANI and HCN, the electronic charge is transferred to the HCN gas from the PANI. This transfer of electron is accepted by the H atom of the HCN gas from the N atom of the PANI forming H–N bonds^38^. Thus, it causes an increase in the resistance of the sensing material when exposed to the HCN gas vapour. HCN molecules also interact with the rGO. The interaction between the HCN and rGO also increases the electrical resistance of the sensors. This increase in the electrical resistance is attributed by the electron-donating property of HCN. When HCN come near to the rGO it increases the hole-type charge on it and shows the p-type semiconductor nature. The oxygen presence in the rGO binds with the hydrogen present in the HCN molecules^35,39^.

## Conclusions

The polymer nanocomposite is synthesized and characterized with FTIR, XRD, TEM and SEM. The TEM micrograph of the graphene shows the formation of sheet structures. The SEM micrograph of PANI shows the formation of a nano-tube of diameter 50 nm and length 250 nm. The PANI is deposited over the entire surface of the MMT. In the case of the PANI/MMT-rGO, the rGO has encapsulated PANI/MMT. The peaks of the XRD pattern confirm the presence of MMT and rGO in the polymer composite. The sensor is exposed to different gases acetone, ammonia, benzene, hydrogen cyanide and xylene. The sensing material can detect HCN gas and give the highest sensing response. The sensing material PANI alone has a low sensing response of 0.05%. The sensing response of the sensing material increases as the MMT and rGO is added to the PANI. The PANI/MMT and PANI/MMT-rGo has the sensitivity of 0.89 ppm^−1^ and 1.1174 ppm^−1^ respectively. In both the sensors we observed that the sensing response of the sensor increases as the concentration of the gas exposed increases. The sensors recovered automatically within 21 s (PANI/MMT) and 25 s (PANI/MMT-rGO). The sensor's performance decreases after 6 months and 8 months.

## Data Availability

The datasets used and/or analysed during the current study available from the corresponding author on reasonable request.
